# Fish Cooking Methods and Impaired Glucose Metabolism Among Japanese Workers: The Furukawa Nutrition and Health Study

**DOI:** 10.3390/nu12061775

**Published:** 2020-06-14

**Authors:** Akiko Nanri, Ayane Takazaki, Takeshi Kochi, Masafumi Eguchi, Isamu Kabe, Tetsuya Mizoue

**Affiliations:** 1 Department of Food and Health Sciences, International College of Arts and Sciences, Fukuoka Women’s University, Fukuoka 813–8529, Japan; a.luv36.k@gmail.com; 2 Department of Epidemiology and Prevention, Center for Clinical Sciences, National Center for Global Health and Medicine, Tokyo 162–8655, Japan; mizoue@hosp.ncgm.go.jp; 3 Department of Health Administration, Furukawa Electric Corporation, Tokyo 100–8322, Japan; takeshi.kochi@furukawaelectric.com (T.K.); masafumi.eguchi@furukawaelectric.com (M.E.); isamu.kabe@kubota.com (I.K.)

**Keywords:** cooking method, fish, impaired glucose metabolism, Japanese, type 2 diabetes

## Abstract

The aim of this study was to examine the cross-sectional association between fish and shellfish intake and impaired glucose metabolism with consideration for cooking methods in a Japanese working population. Participants were 1774 workers aged 18–69 years. Dietary intake was assessed using a validated self-administered diet history questionnaire. Participants were asked about their most frequently used cooking method for fish, and the method was classified as either “raw and stewing” or “broiling, deep-frying, and stir-frying”. Impaired glucose metabolism was defined by a history of diabetes, current use of anti-diabetic drugs, fasting blood glucose ≥110 mg/dl, or HbA1c ≥6.0%. Logistic regression analysis was used to estimate the odds ratios of impaired glucose metabolism for fish intake by cooking method. Fish intake was not associated with impaired glucose metabolism in either group. When the outcome was defined as diabetes, the odds of diabetes increased with fish intake among participants who most frequently used broiling, deep-frying, or stir-frying methods, albeit they were not statistically significant; the multivariable-adjusted odds ratio for the highest versus the lowest tertiles of fish intake was 1.95 (95%CI, 0.71–5.41). Cooking methods for fish may not modify the association between fish intake and impaired glucose metabolism among Japanese populations.

## 1. Introduction

The global prevalence of diabetes and impaired glucose tolerance in adults has increased over recent decades [1]. Data from the International Diabetes Federation estimated that there were 451 million people with diabetes worldwide in 2017 and predicted that this will increase to 693 million by 2045 [1]. According to the National Health and Nutrition Survey, Japan, in 2017 [2], 18.1% of men and 10.5% of women in Japan aged 20 or above have diabetes. This incidence increases with age, reaching 25.7% and 19.8% for men and women aged 70 or above, respectively. Diabetes and its associated complications reduce quality of life and are a major healthcare burden, making it of paramount importance to seek and develop strategies to prevent diabetes.

Dietary factors have received considerable attention as modifiable factors that can affect type 2 diabetes risk. An ecological study of 41 countries suggested that high fish and seafood intake may reduce type 2 diabetes risk [3]. However, the association between fish intake and type 2 diabetes based on prospective studies is controversial. According to a meta-analysis of 16 studies [4], the association differed by geographical region: fish intake was associated with increased risk of type 2 diabetes in US studies; no association was seen in European studies; and an inverse association was seen in Asian/Australian studies. These differences in association might be partly explained by regional differences in the amount of fish consumed, cooking methods for fish and shellfish, and overall dietary pattern. Regarding cooking methods, for example, while Japanese populations traditionally consume raw fish (e.g., *sashimi* and *sushi*), this is rare among other populations. In contrast, Western populations commonly consume broiled fish and fried fish. Previously, only three studies have examined the association between intake of fried fish or broiled fish and type 2 diabetes risk in Western populations [5,6,7]. However, no studies have examined the association of fish intake with type 2 diabetes based on cooking methods such as raw (no cooking) and stewing. Further, no study has yet investigated the association of cooking methods for fish with type 2 diabetes in Asia, including Japan. In this study, we examined the association between cooking methods for fish and type 2 diabetes. Specifically, we examined the association of fish and shellfish intake with impaired glucose metabolism with consideration for cooking methods among Japanese workers.

## 2. Materials and Methods

### 2.1. Study Procedure

As part of the Japan Epidemiology Collaboration of Occupation Health Study, the Furukawa Nutrition and Health Study, a nutritional epidemiological survey was conducted at the time of the regular health examination among workers of a manufacturing company and its affiliated companies (electrical machinery and apparatus manufacturing) in Chiba and Kanagawa Prefectures, Japan, in April 2012 and May 2013. Prior to the health checkup, we asked all employees to participate in the survey and to complete two types of survey questionnaire, one specifically designed for diet and the second for overall health-related lifestyle. Of the 2828 checkup attendees, 2162 participants (1930 men and 232 women aged 18–79 years) agreed to participate in the survey, giving a response rate of 76%. On the day of the health checkup, research staff checked the questionnaire for completeness and, where necessary, clarified responses with the participants. Participants were also asked to donate 7 mL of venous blood. Additionally, we obtained health checkup data including the results of anthropometric and biochemical measurements and information on history of diseases. The study protocol was approved by the Ethics Committee of the National Center for Global Health and Medicine, Japan. Written informed consent was obtained from all participants prior to the survey.

### 2.2. Participants

**Figure 1:**
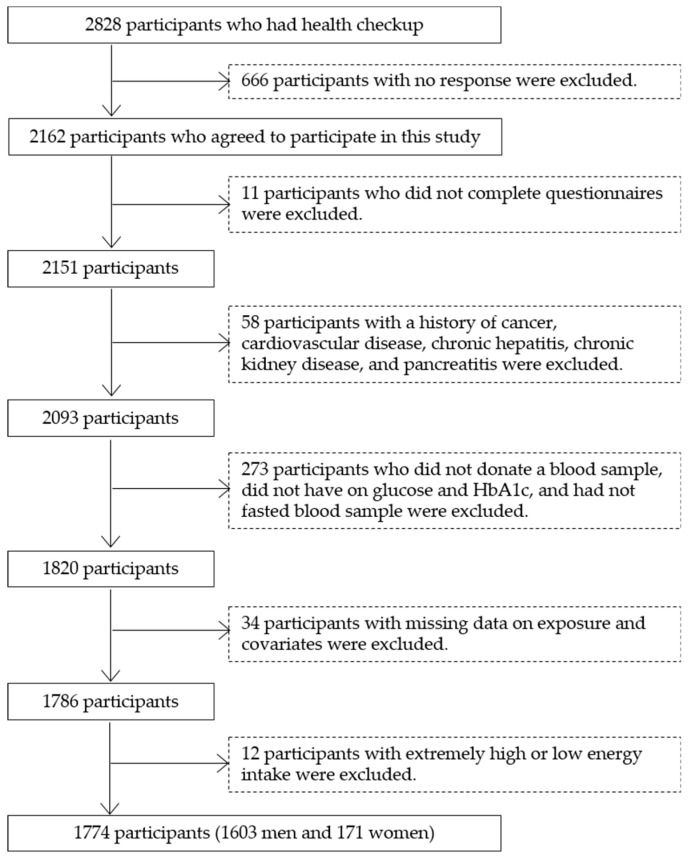
Flowchart of the participants.

Of the 2162 participants who participated in the survey, 11 participants who did not complete either the diet or health-related lifestyle questionnaire were excluded (Figure 1). Additionally, 58 participants who reported a history of cancer, cardiovascular disease, chronic hepatitis, chronic kidney disease including nephritis, and pancreatitis were also excluded. Of the remaining 2093 participants, 273 participants who did not donate a blood sample (*n* = 207), did not have data on glucose and HbA1c (*n* = 2), and had not fasted for at least 10 h prior to the examination (*n* = 64) were also excluded. Additionally, 34 participants with missing data on exposure (fish cooking method) and covariates used in the present analysis were also excluded. Finally, those with extremely high or low energy intake (exceeding mean ± 3 SD (standard deviations)) were excluded, leaving 1774 participants (1603 men and 171 women) for analysis.

### 2.3. Blood Measurements

As part of the health checkup, the plasma glucose concentration was enzymatically assayed using Quick-auto-neo-GLU-HK (Shino-Test Corp., Tokyo, Japan), and HbA1c levels were measured by latex agglutination immunoassay using the Determiner HbA1c kit (Kyowa Medex Co., Ltd., Tokyo, Japan) at an external laboratory (Kinki Kenko Kanri Center, Shiga, Japan).

### 2.4. Ascertainment of Impaired Glucose Metabolism

Impaired glucose metabolism, the primary outcome, was defined by a history of diabetes, current use of anti-diabetic drugs, fasting blood glucose ≥110 mg/dl, or HbA1c ≥ 6.0% with reference to the repot of the Committee of the Japan Diabetes Society on the Diagnostic Criteria of Diabetes Mellitus [8]. Diabetes, the secondary outcome, was defined by a history of diabetes, current use of anti-diabetic drugs, fasting blood glucose ≥126 mg/dl, or HbA1c ≥ 6.5% [8].

### 2.5. Dietary Assessment

Information on cooking methods for fish and shellfish was obtained using the questionnaire for health-related lifestyle. Participants were asked to select the most frequently used cooking method for fish and shellfish from 5 options (raw, stewing, broiling, deep-frying and stir-frying). Dietary habits during the preceding month were assessed using a validated brief self-administered diet history questionnaire (BDHQ) [9], which consists of five sections: (1) frequency of intake of 46 foods and non-alcoholic beverages; (2) daily frequency of rice and miso soup intake; (3) frequency of alcohol consumption and the frequency of consuming five alcoholic beverages per typical drinking occasion; (4) usual cooking methods; and (5) dietary behavior. Dietary intake of 58 food and beverage items, energy, and selected nutrients were estimated using an ad hoc computer algorithm for the BDHQ, with reference to the Standard Tables of Food Composition in Japan [10]. According to the validation study for the BDHQ, which used 16-day weighed dietary records as the gold standard, Spearman’s correlation coefficient for energy-adjusted food group intake in 92 men and 92 women aged 31–76 years ranged from 0.21 for potatoes to 0.83 for alcoholic beverages in men and 0.14 for fruit and vegetable juice to 0.82 for alcoholic beverages in women [9].

### 2.6. Other Variables

The questionnaire also examined night and rotating shift work, smoking, alcohol consumption, physical activity during work and housework or while commuting to work, and leisure-time physical activity. Physical activity during work and housework or while commuting and leisure-time were expressed as the sum of metabolic equivalents (METs) multiplied by the duration (in hours) across all levels of physical activity. Body height was measured to the nearest 0.1 cm while the participant was standing without shoes. Body weight in light clothes was measured to the nearest 0.1 kg. BMI was calculated as weight in kilograms divided by the square of height in meters.

### 2.7. Statistical Analysis

First, we divided participants into two groups based on the most frequently used cooking method for fish and shellfish: “raw and stewing” (cooking methods that do not use oil) and “broiling, deep-frying, and stir-frying” (cooking methods that use high temperatures and oil). Second, we divided participants in each group into tertiles of fish and shellfish intake (<26.2, 26.2 -< 41.3, and ≥41.3 g/1000 kcal for raw and stewing; <25.8, 25.8 -< 39.95, and ≥39.95 g/1000 kcal for broiling, deep-frying, and stir-frying). Data were expressed as mean (standard deviation) for continuous variables and percentages for categorical variables. Trend associations between confounding factors and fish and shellfish intake were analyzed using linear regression analysis for continuous variables and the Mantel–Haenszel chi-squared test for categorical variables, with the median intake in each tertile of fish and shellfish intake as a continuous variable (independent variable).

Multiple logistic regression was performed to estimate the odds ratios and 95% CIs of impaired glucose metabolism for tertiles of total fish and shellfish intake, with the lowest tertile category as a reference, by cooking method. The first model was adjusted for age (year, continuous), sex, and site (survey in April 2012 or in May 2013). The second model was further adjusted for night or rotating shift work (yes or no); smoking (never-smoker, quitter, current smoker consuming <20 cigarettes/day, or current smoker consuming ≥20 cigarettes/day); alcohol consumption (nondrinker, or drinker consuming <23 g, 23 to <46 g, or ≥46 g of ethanol/day); leisure-time physical activity (MET-hours/week, quartile); physical activity at work and housework or while commuting to work (MET-hours/day, quartile); parental history of diabetes (yes or no); history of hypertension (yes or no); history of dyslipidemia (yes or no); total energy intake (kcal/day); energy-adjusted intake of rice (g/day), vegetables (g/day), fruit (g/day), meat (g/day), calcium (mg/day), magnesium (mg/day), dietary fiber (g/day), saturated fatty acids (% energy), monounsaturated fatty acids (% energy), polyunsaturated fatty acids (% energy), protein (% energy), and sugar (g/day); and coffee consumption (g/day). These dietary factors have been reported to be associated with type 2 diabetes [11,12,13,14,15,16,17]. The final model was further adjusted for BMI (kg/m^2^, continuous). Trend associations were assessed by treating the median intake in each tertile of fish and shellfish intake as a continuous variable. The interaction term obtained by multiplying fish and shellfish intake (g/day, continuous) with the cooking method group (“raw and stewing” and “broiling, deep-frying, and stir-frying”) was added to the model to assess statistical interactions. *p* values for interaction <0.1 were regarded as statistically significant. In the present study, since the number of women was small (10%), the association was examined among men only. In addition, multiple linear regression was performed to estimate the means and 95% CIs of fasting glucose and HbA1c for tertiles of total fish and shellfish intake by cooking method with adjustment for covariates as mentioned above.

Moreover, to examine whether the prevalence of impaired glucose metabolism differed according to major cooking methods for fish and shellfish, we estimated odds ratios and 95% CIs of impaired glucose metabolism for each of the cooking methods using multiple logistic regression analysis. In this analysis, we used the broiling category as a reference because the number of participants who reported broiling was greatest among the cooking methods for fish and shellfish (73.8%). In addition, deep-frying and stir-frying were combined because few participants indicated these responses as the most frequently used cooking method for fish and shellfish (*n* = 16 for deep-frying and *n* = 20 for stir-frying). Therefore, participants were classified into four categories of cooking methods (“raw,” “stewing”, “broiling”, and “deep-frying and stir-frying”). The adjusted model was the same as that described above, but fish intake (g/1000 kcal) was additionally adjusted in the second model. As sensitivity analysis, we repeated the above analysis among participants who consumed fish and shellfish at the median intake or above (≥32 g/1000 kcal) for the study population. Two-sided *p* values < 0.05 were regarded as statistically significant. All analyses were performed using Statistical Analysis System (SAS) software version 9.3 (SAS Institute, Cary, NC, USA).

## 3. Results

The number of participants by the most frequently used cooking method for fish and shellfish was 250 (14.1%) for raw, 178 (10.0%) for stewing, 1310 (73.8%) for broiling, 16 (0.9%) for deep-frying, and 20 (1.1%) for stir-frying. The characteristics of the study participants according to tertiles of fish and shellfish intake by the most frequently used cooking method are shown in Table 1. In both groups, participants with higher intake of fish and shellfish tended to be older and to consume less rice and more vegetables, fruit, calcium, magnesium, dietary fiber, monounsaturated fatty acids, polyunsaturated fatty acids, and protein than those with lower intake. In addition, participants with higher intake of fish in the “raw and stewing” group were less likely to be shift workers, and those with higher intake of fish in the “broiling, deep-frying, and stir-frying” group were less likely to be current smokers.

In total, 196 (11.0%) and 87 (4.9%) participants were identified as having impaired glucose metabolism and diabetes, respectively. The odds ratios of impaired glucose metabolism according to fish and shellfish intake for the two groups (“raw and stewing” and “broiling, deep-frying, and stir-frying”) are shown in Table 2. The association between fish and shellfish intake and impaired glucose metabolism was not statistically significant in either group. Among participants who reported broiling, deep-frying, and stir-frying as the most frequently used cooking methods for fish and shellfish, the multivariable-adjusted odds ratios for impaired glucose metabolism of the lowest through to the highest tertile of fish and shellfish intake were 1.00 (reference), 1.00 (95% CI, 0.58–1.72), and 1.20 (95% CI, 0.60–2.40) (trend *p* = 0.58). Among participants who reported raw (no cooking) and stewing as the most frequently used cooking methods for fish and shellfish, the corresponding multivariable-adjusted odds ratios were 1.00 (reference), 0.95 (95% CI, 0.32–2.78), and 0.64 (95% CI, 0.16–2.54) (trend *p* = 0.52). There was no significant interaction between fish and shellfish intake and cooking method (interaction *p* = 0.46). When the outcome was defined as diabetes, the odds of diabetes increased in the highest tertile of fish and shellfish intake among participants who preferred broiling, deep-frying, and stir-frying; the multivariable-adjusted odds ratio for diabetes of the highest versus lowest tertile of fish and shellfish intake was 1.95 (95% CI, 0.71–5.41) (trend *p* = 0.16). Among those who preferred raw and stewing, such association was not observed (interaction *p* = 0.078). When we examined the association among men only, the findings were not materially change.

The association between fasting glucose and HbA1c and fish and shellfish intake by fish cooking methods is shown in Table 3. Among participants who reported raw and stewing as the most frequently used cooking methods for fish and shellfish, fish and shellfish intake was inversely associated with HbA1c; the multivariable-adjusted mean of HbA1c for the lowest through highest tertile of fish and shellfish intake were 5.58% (95% CI, 5.49–5.67), 5.52% (95%CI, 5.45–5.60), 5.39% (95%CI, 5.29–5.49) (trend *p* = 0.02). Among participants who most frequently used broiling, deep-frying, or stir-frying methods, fish and shellfish intake was not associated with fasting glucose and HbA1c.

For the association between cooking methods for fish and shellfish and impaired glucose metabolism, the odds ratio of impaired glucose metabolism among participants in the “deep-frying and stir-frying” category was higher than that of participants in the “broiling” category, although the association was not statistically significant. The multivariable-adjusted odds ratio for impaired glucose metabolism was 1.62 (95% CI, 0.47–5.58) in the “deep-frying and stir-frying” category compared to the “broiling” category after adjustment for covariates including fish and shellfish intake and BMI (data not shown in table). In sensitivity analysis, where we restricted the analysis to participants who consumed fish and shellfish at the median intake or above for the study population, the higher odds ratio for impaired glucose metabolism associated with deep-frying and stir-frying was further strengthened (odds ratio 2.61; 95% CI, 0.49–14.07) (data not shown in table).

## 4. Discussion

In this cross-sectional study of Japanese workers, we observed no significant association between fish and shellfish intake and impaired glucose metabolism for either of the two groups of frequently used cooking methods. However, the odds ratio for impaired glucose metabolism tended to increase with fish and shellfish intake in the “broiling, deep-frying, and stir-frying” group, but tended to decrease with fish and shellfish intake in the “raw and stewing” group. In addition, the category “deep-frying and stir-frying” was associated with increased prevalence of impaired glucose metabolism compared with “broiling”, although this increase was not statistically significant. To our knowledge, this is the first study to examine the association of cooking methods for fish, including raw (no cooking) and stewing, with impaired glucose metabolism.

Previously, two studies examined the association of fried fish intake with type 2 diabetes risk [6,7]. In the Cohort of Swedish Men study [7], fried fish intake was associated with increased risk of type 2 diabetes, but total fish intake was not. In the European Prospective Investigation of Cancer (EPIC)-Norfolk cohort study [6], total fish intake was associated with decreased risk of type 2 diabetes, but fried fish intake was not. In these previous studies [6,7], participants were divided into two or five categories according to fried fish intake, and the association of fried fish intake with type 2 diabetes was subsequently examined. In contrast, in the present study, we divided participants into two groups based the most frequently used cooking method for fish and examined the association of total fish intake (not fish intake by each cooking method) with impaired glucose metabolism for each of the two groups of cooking methods. Therefore, the methods of analysis used were different between ours and the previous studies. However, the non-significant positive association between fish intake and impaired glucose metabolism among participants in the “broiling, deep-frying, and stir-frying” group may be supported by the previous findings. Moreover, no study has previously examined the association of raw (no cooking) and stewing methods for cooking fish with type 2 diabetes. Therefore, our finding of an inverse association between fish intake and impaired glucose metabolism among participants in the “raw and stewing group” provides new insight into the association between fish intake and type 2 diabetes.

While the mechanism linking deep-frying and stir-frying methods for cooking fish to impaired glucose metabolism is unclear, there are several possible explanations. Absorption of fat during frying produces more energy-dense foods and may contribute to higher overall fat intake. Indeed, total energy intake was higher among participants in the “deep-frying and stir-frying” category than those in the other categories, although the difference was not statistically significant (mean (SD): 1845 (450) kcal for “deep-frying and stir-frying”, 1807 (527) kcal for “raw”, 1812 (457) kcal for “stewing”, and 1798 (478) kcal for “broiling”). In addition, total fat intake was higher among participants in the “deep-frying and stir-frying” category (25.2% of energy for “deep-frying and stir-frying” category, 23.5% of energy for “raw”, 23.7% of energy for “stewing”, and 24.0% of energy for “broiling”). Moreover, the fatty acid composition of foods changes with frying, and frying fish may lead to a loss of long-chain omega-3 fatty acids and an increase in other fatty acids depending on the type of fat used for frying [18]. Further, high-temperature cooking, such as frying, induces the formation of advanced glycation end products, which may contribute to insulin resistance [19]. Frying may also contribute to the formation of mutagenic compounds, such as heterocyclic amines [20]. Therefore, the beneficial effects of fish on type 2 diabetes might be negated by frying.

Strengths of the present study include its high participation rate, use of a validated questionnaire for diet, and adjustment for known and suspected risk factors of type 2 diabetes. Because this study was conducted among employees of a selected company during nonselective recruitment for the annual health checkup and had a high study participation rate, the possibility of bias associated with selective study participation is low. Moreover, our sample of the Japanese population allowed us to examine various cooking methods for fish and shellfish including raw (no cooking), stewing, broiling, deep-frying, and stir-frying. Our study also had some limitations. First, an association derived from a cross-sectional study does not necessarily indicate causality. Second, the statistical power may not have been sufficient to detect a significant association. In particular, few participants reported deep-frying and stir-frying as the most frequently used cooking methods for fish and shellfish. The present findings should be interpreted with caution. Third, we assessed dietary intake using a self-administered questionnaire and at only one time point, and the validity of fish and shellfish intake, assessed using the BDHQ, was relatively low [9]. Therefore, misclassification may have occurred in the assessment of cooking methods and fish and shellfish intake. Fourth, as mentioned above, we examined the association of total fish intake (not fish intake according to each cooking method) with impaired glucose metabolism based on the most frequently used cooking method. Although we did ask participants about the frequency of fish intake for each cooking method using the BDHQ, we did not use this data because fish intake for these cooking methods has not been validated. Fifth, the type of oil (e.g., plant, animal, or synthetic) used by participants might affect the present results. However, we did not collect information on type of oil. Sixth, although we adjusted for important risk factors for impaired glucose metabolism, we cannot rule out the possibility of bias due to unrecognized confounders or residual confounding. Finally, because the study participants were workers of a selected company and most of them (90%) were men, the present findings may not be applicable to a general population.

## 5. Conclusions

We did not observe a significant association between cooking methods for fish and shellfish and impaired glucose metabolism. However, the prevalence of impaired glucose metabolism tended to increase with fish and shellfish intake among participants who most frequently used broiling, deep-frying, and stir-frying but tended to decrease with fish and shellfish intake among those who most frequently used raw (no cooking) and stewing methods. Because few studies have examined the association of cooking methods for fish with diabetes, and the present study was not designed to examine the association between intake of fish cooked by each of the examined methods and impaired glucose metabolism, further studies are needed to examine these associations.

## Figures and Tables

**Table 1:** Characteristics of participants according to tertiles of fish and shellfish intake by fish cooking methods.

	Raw and Stewing				Broiling, Deep-Frying, and Stir-Frying			
	Tertile of Fish and Shellfish Intake				Tertile of Fish and Shellfish Intake			
	Lowest	Middle	Highest	Trend *p* ^1^	Lowest	Middle	Highest	Trend *p* ^1^
No. of participants	142	143	143		448	449	449	
Age, year, mean (standard deviation)	43.0 (9.6)	43.9 (9.2)	46.6 (10.3)	0.001	42.4 (8.4)	43.3 (8.9)	44.8 (9.4)	<0.001
Women, %	7.7	11.2	11.2	0.38	8.0	9.6	10.9	0.15
Site A, %	48.6	53.1	35.0	0.010	44.9	46.5	36.1	0.004
BMI, kg/m^2^, mean (standard deviation)	23.7 (3.6)	23.2 (3.5)	23.9 (3.4)	0.53	23.2 (3.5)	23.3 (3.1)	23.3 (3.2)	0.79
Night and rotating shift work, %	27.5	17.5	14.7	0.010	20.3	17.4	19.4	0.82
Current smokers, %	38.7	26.6	28.7	0.10	31.7	27.6	24.9	0.028
High physical activity ^2^, %	40.8	28.0	30.1	0.09	37.3	28.3	34.1	0.49
High leisure-time physical activity ^3^, %	34.5	31.5	34.3	0.97	30.4	35.6	34.1	0.31
Alcohol consumption ≥1 day/week, %	65.5	60.1	62.9	0.74	48.7	55.7	52.8	0.31
Parental history of diabetes, %	14.1	16.1	11.9	0.52	16.5	15.4	16.3	0.96
History of hypertension, %	12.7	11.2	14.7	0.55	9.2	8.5	9.6	0.78
History of dyslipidemia, %	6.3	7.0	8.4	0.50	4.0	5.1	4.5	0.82
Dietary intake, mean (standard deviation)								
Total energy, kcal	1830 (525)	1770 (495)	1826 (476)	0.94	1788 (468)	1769 (457)	1841 (505)	0.067
Rice, g/1000 kcal	193 (78)	181 (67)	161 (67)	<0.001	201 (69)	185 (67)	169 (63)	<0.001
Fish and shellfish, g/1000 kcal	18 (6)	32 (4)	59 (21)	<0.001	18 (6)	32 (4)	57 (17)	<0.001
Meat, g/1000 kcal	41 (19)	37 (16)	39 (18)	0.35	37 (18)	38 (16)	39 (18)	0.10
Vegetables, g/1000 kcal	92 (46)	116 (61)	132 (80)	<0.001	103 (67)	118 (54)	137 (67)	<0.001
Fruits, g/1000 kcal	31 (40)	44 (53)	51 (54)	0.001	40 (50)	46 (50)	50 (49)	0.003
Calcium, mg/1000 kcal	189 (78)	230 (85)	256 (103)	<0.001	203 (82)	236 (75)	271 (85)	<0.001
Magnesium, mg/1000 kcal	111 (21)	125 (23)	137 (35)	<0.001	113 (24)	126 (20)	140 (26)	<0.001
Dietary fiber, g/1000 kcal	4.8 (1.3)	5.6 (1.8)	5.9 (2.2)	<0.001	5.3 (1.8)	5.7 (1.5)	6.2 (1.8)	<0.001
Saturated fatty acids, % energy	5.9 (1.9)	6.0 (2.1)	6.1 (1.5)	0.55	5.9 (1.8)	6.2 (1.7)	6.3 (1.6)	<0.001
Monounsaturated fatty acids, % energy	8.4 (2.2)	8.5 (2.1)	8.9 (2.0)	0.03	8.3 (2.2)	8.7 (2.1)	9.1 (2.0)	<0.001
Polyunsaturated fatty acids, % energy	5.6 (1.4)	6.0 (1.4)	6.4 (1.4)	<0.001	5.7 (1.4)	6.1 (1.3)	6.6 (1.3)	<0.001
Protein, % energy	12.0 (2.0)	13.2 (1.7)	15.5 (2.6)	<0.001	12.1 (1.9)	13.6 (1.8)	15.6 (2.3)	<0.001
Sugar, g/1000kcal	4.4 (4.1)	3.9 (3.4)	3.2 (2.4)	0.002	4.2 (3.8)	3.8 (3.7)	3.8 (3.3)	0.16
Coffee consumption ≥1 cup/day, %	68.3	65.7	58.7	0.08	67.6	68.2	64.4	0.26

^1^ Based on Mantel–Haenszel chi-squared test for categorical variables and linear regression analysis for continuous variables, assigning median value for each tertile of fish and shellfish intake. ^2^ Physical activity during work and housework or while commuting ≥12.5 METs-hour/day in raw and stewing group and ≥11.75 METs-hour/day in broiling, deep-frying, and stir-frying group (the highest tertile). ^3^ Leisure-time physical activity ≥7.0 METs-hour/week in both groups (the highest tertile).

**Table 2:** Odds ratios (95% CI) of impaired glucose metabolism according to tertiles of fish and shellfish intake by fish cooking methods.

	Tertile of Fish and Shellfish Intake			
	Lowest	Middle	Highest	Trend *p* ^1^
Raw and stewing				
Fish intake (median, g/1000 kcal)	19	32.1	52.5	
Fish intake (range, g/1000kcal)	<26.2	26.2-<41.3	≥41.3	
No. of participants	142	143	143	
Impaired glucose metabolism				
No. of cases	15	13	19	
Adjusted^2^ OR (95% CI)	1.00 (ref)	0.84 (0.37–1.93)	0.96 (0.44–2.10)	0.97
Adjusted^3^ OR (95% CI)	1.00 (ref)	0.87 (0.32–2.39)	0.78 (0.21–2.83)	0.70
Adjusted^4^ OR (95% CI)	1.00 (ref)	0.95 (0.32-2.78)	0.64 (0.16–2.54)	0.52
Diabetes				
No. of cases	9	7	8	
Adjusted ^2^ OR (95% CI)	1.00 (ref)	0.74 (0.26-2.12)	0.73 (0.26-2.06)	0.58
Adjusted ^3^ OR (95% CI)	1.00 (ref)	0.83 (0.20–3.50)	1.16 (0.17–7.87)	0.89
Adjusted ^4^ OR (95% CI)	1.00 (ref)	1.27 (0.24–6.81)	0.89 (0.11–7.32)	0.92
Broiling, deep-frying, and stir-frying				
Fish intake (median, g/1000 kcal)	18.8	32.0	51.9	
Fish intake (range, g/1000kcal)	<25.8	25.8 -<39.95	≥39.95	
No. of participants	448	449	449	
Impaired glucose metabolism				
No. of cases	43	46	60	
Adjusted ^2^ OR (95% CI)	1.00 (ref)	0.99 (0.63–1.55)	1.26 (0.82–1.94)	0.25
Adjusted ^3^ OR (95% CI)	1.00 (ref)	0.98 (0.58–1.65)	1.21 (0.62–2.38)	0.53
Adjusted ^4^ OR (95% CI)	1.00 (ref)	1.00 (0.58–1.72)	1.20 (0.60–2.40)	0.58
Diabetes				
No. of cases	18	15	30	
Adjusted ^2^ OR (95% CI)	1.00 (ref)	0.80 (0.39–1.62)	1.44 (0.78–2.67)	0.16
Adjusted ^3^ OR (95% CI)	1.00 (ref)	0.97 (0.42–2.28)	1.88 (0.70–5.02)	0.17
Adjusted ^4^ OR (95% CI)	1.00 (ref)	1.07 (0.44–2.62)	1.95 (0.71–5.41)	0.16

Abbreviation: CI, confidence interval; OR, odds ratio; ref, reference. ^1^ Based on multiple logistic regression analysis with assignment of median value for each tertile. ^2^ Adjusted for age (year), sex, and site (survey in April 2012 or in May 2013). ^3^ Additionally adjusted for night or rotating shift work (yes or no), smoking status (never-smoker, quitter, current smoker consuming <20 cigarettes/day, or current smoker consuming ≥20 cigarettes/day), alcohol drinking (nondrinker or drinker consuming <23 g, 23-< 46 g, or ≥46 g of ethanol/day), leisure-time physical activity (METs-hour/week, quartile), physical activity during work and housework or while commuting to work (METs-hour/day, quartile), parental history of diabetes (yes or no), history of hypertension (yes or no), history of dyslipidemia (yes or no), total energy intake (kcal/day), energy-adjusted intakes of rice (g/day), vegetables (g/day), fruits (g/day), meat (g/day), calcium (mg/day), magnesium (mg/day), dietary fiber (g/day), saturated fatty acids (% energy), monounsaturated fatty acids (% energy), polyunsaturated fatty acids (% energy), protein (% energy), and sugar (g/day), and coffee consumption (g/day). ^4^ Additionally adjusted for BMI (kg/m^2^).

**Table 3:** Adjusted means (95% CI) of fasting glucose and HbA1c according to tertiles of fish and shellfish intake by fish cooking methods.

	Tertile of Fish and Shellfish Intake			
	Lowest	Middle	Highest	Trend *p* ^1^
Raw and stewing				
Fasting glucose (mg/dl)				
Adjusted ^2^ mean (95% CI)	93.2 (91.2–95.3)	92.0 (90.0–94.1)	93.5 (91.5–95.6)	0.75
Adjusted ^3^ mean (95% CI)	93.8 (91.4–96.2)	91.9 (89.8–94.0)	93.2 (90.5–95.8)	0.80
Adjusted ^4^ mean (95% CI)	94.0 (91.6-96.3)	92.3 (90.3–94.2)	92.6 (90.1–95.1)	0.51
HbA1c (%)				
Adjusted ^2^ mean (95% CI)	5.52 (5.44–5.61)	5.50 (5.42–5.59)	5.47 (5.39–5.56)	0.43
Adjusted ^3^ mean (95% CI)	5.57 (5.48–5.67)	5.51 (5.42–5.59)	5.42 (5.32–5.53)	0.07
Adjusted ^4^ mean (95% CI)	5.58 (5.49–5.67)	5.52 (5.45–5.60)	5.39 (5.29–5.49)	0.02
Broiling, deep-frying, and stir-frying				
Fasting glucose (mg/dl)				
Adjusted ^2^ mean (95% CI)	92.2 (91.0–93.4)	91.6 (90.4–92.8)	92.6 (91.4–93.9)	0.57
Adjusted ^3^ mean (95% CI)	92.6 (91.1–94.1)	91.7 (90.5–92.9)	92.1 (90.5–93.7)	0.72
Adjusted ^4^ mean (95% CI)	92.7 (91.3-94.1)	91.7 (90.5–92.9)	92.1 (90.5–93.6)	0.65
HbA1c (%)				
Adjusted ^2^ mean (95% CI)	5.47 (5.42–5.52)	5.45 (5.40–5.50)	5.48 (5.43–5.53)	0.65
Adjusted ^3^ mean (95% CI)	5.48 (5.43–5.54)	5.46 (5.41–5.51)	5.46 (5.40–5.52)	0.67
Adjusted ^4^ mean (95% CI)	5.49 (5.43–5.54)	5.46 (5.41–5.50)	5.46 (5.40–5.52)	0.60

Abbreviation: CI, confidence interval. ^1^ Based on multiple linear regression analysis with assignment of median value for each tertile. ^2^ Adjusted for age (year), sex, and site (survey in April 2012 or in May 2013). ^3^ Additionally adjusted for night or rotating shift work (yes or no), smoking status (never-smoker, quitter, current smoker consuming <20 cigarettes/day, or current smoker consuming ≥20 cigarettes/day), alcohol drinking (nondrinker or drinker consuming <23 g, 23-<46 g, or ≥46 g of ethanol/day), leisure-time physical activity (METs-hour/week, quartile), physical activity during work and housework or while commuting to work (METs-hour/day, quartile), parental history of diabetes (yes or no), history of hypertension (yes or no), history of dyslipidemia (yes or no), total energy intake (kcal/day), energy-adjusted intakes of rice (g/day), vegetables (g/day), fruits (g/day), meat (g/day), calcium (mg/day), magnesium (mg/day), dietary fiber (g/day), saturated fatty acids (% energy), monounsaturated fatty acids (% energy), polyunsaturated fatty acids (% energy), protein (% energy), and sugar (g/day), and coffee consumption (g/day). ^4^ Additionally adjusted for BMI (kg/m^2^).
